# Bone regeneration of induced pluripotent stem cells derived from peripheral blood cells in collagen sponge scaffolds

**DOI:** 10.1590/1678-7757-2021-0491

**Published:** 2022-02-21

**Authors:** Hiroshi Kato, Katsuhito Watanabe, Akiko Saito, Shoko Onodera, Toshifumi Azuma, Masayuki Takano

**Affiliations:** 1 Tokyo Dental College Department of Oral and Maxillofacial Surgery Tokyo Japan Tokyo Dental College, Department of Oral and Maxillofacial Surgery, Tokyo, Japan.; 2 The Jikei University School of Medicine Department of Dentistry Tokyo Japan The Jikei University School of Medicine, Department of Dentistry, Tokyo, Japan.; 3 Tokyo Dental College Department of Biochemistry Tokyo Japan Tokyo Dental College, Department of Biochemistry, Tokyo, Japan.

**Keywords:** Induced pluripotent stem cell, Osteoblast, Bone regeneration

## Abstract

Stem cell-based regeneration therapy offers new therapeutic options for patients with bone defects because of significant advances in stem cell research. Although bone marrow mesenchymal stem cells are the ideal material for bone regeneration therapy using stem cell, they are difficult to obtain. Induced pluripotent stem cells (iPSCs) are now considered an attractive tool in bone tissue engineering. Recently, the efficiency of establishing iPSCs has been improved by the use of the Sendai virus vector, and it has become easier to establish iPSCs from several type of somatic cells. In our previous study, we reported a method to purify osteogenic cells from iPSCs.

**Objective:**

This study aimed to evaluate the osteogenic ability of iPSCs derived from peripheral blood cells.

**Methodology:**

Mononuclear cells (MNCs) were obtained from human peripheral blood. Subsequently, T cells were selectively obtained from these MNCs and iPSCs were established using Sendai virus vectors. Established iPSCs were evaluated by the expression of undifferentiated markers and teratoma formation assays. Osteoblasts were induced from these iPSCs and evaluated by the expression of osteoblast markers. Additionally, the induced osteoblasts were transplanted into rat critical size calvaria bone defect models with collagen sponge scaffolds. Samples were evaluated by radiographical and histological assessments.

**Results:**

Induced osteoblasts expressed several osteoblast-specific markers. The results of radiographical and histological assessments revealed that the cell transplant group had bone formations superior to those of the control group.

**Conclusions:**

This study suggests that peripheral blood MNCs have the potential to differentiate into osteoblasts. Although there are some hurdles in iPSC transplantation, osteoblasts obtained from MNC-iPSCs could be applied to bone regeneration therapy in the future.

## Introduction

In the oral and maxillofacial region, bone grafts are required for patients with congenital diseases, such as a cleft lip or palate, and for patients who have acquired bone defects resulting from surgical procedures.^1-5^

Autogenous bone grafting remains the “gold standard” for extensive bone reconstructions because of the osteogenic, osteoinductive, and osteoconductive properties of this method.^2^ However, the procedures to harvest extra-oral autogenous bone grafts, such as from the calvaria, iliac, tibia, and fibula, may include complications, including donor site morbidity, pain, rejection, infection, and a limited amount of graft tissue.^6^ Therefore, there are expectations that stem cells may enable a new form of bone regenerative therapy instead of the autogenous bone graft treatment.

It has been reported that regenerative therapy can be achieved by stem cells, scaffolding, and cytokines. In recent studies on this topic, the interaction between stem cells and biomaterials has emerged as a topic of interest, and it has been found that the secretome produced by stem cells has various physiological and biological effects in stem cell transplantation. In addition, biomaterials play a major role in stem cell prosperity. The current strategy is to create new scaffolds for stem cell transplantation based on computer-aided design (CAD) technology.^7^ Stem cells are identified according to their source, for example, bone marrow mesenchymal stem cells (MSCs), adipo-derived MSCs, perivascular stem cells, and induced pluripotent stem cells (iPSCs).^8^

In recent decades, potential advantages of the use of MSCs in bone tissue regeneration studies and clinical experiments have been reported.^8,9^ However, their availability and self-renewal capacity are limited and are significantly affected by age. To overcome this hurdle, iPSCs have been proposed as ideal cells due to their versatile differentiation capacity.^10^ Several different human somatic cells have been reprogrammed into iPSCs.^11-13^ iPSCs have often established using fibroblasts; however, the efficiency of establishing iPSCs has been improved by the use of the Sendai virus vector, making it is relatively easy to establish iPSCs from blood cells. Consequently, it is not necessary to collect tissues using a surgical technique; only peripheral blood collection is required.

Furthermore, iPSCs can be induced from a variety of patient-specific cells and can be subsequently used to explore disease mechanisms. Moreover, iPSCs can be used as novel therapeutic molecular targets for drug development and regenerative cell therapies.^14-17^ We have previously reported an effective procedure to generate osteoblastic cells from human iPSCs.^18^ However, the study used an iPSC cell line derived from fibroblasts, and it was unknown if it would be possible to induce osteogenic cells from peripheral blood cells.

Here, we generated iPSCs derived from peripheral blood mononuclear cells (MNCs) and demonstrated their osteogenic potential *in vitro* and *in vivo* in a rat calvaria bone defect model. Peripheral blood mononuclear cells (PBMCs), including monocytes, T cells, B cells, and NK cells, are blood cells with spherical nuclei. This study indicates that peripheral MNCs can serve as a source of stem cells that can be differentiated effectively into active osteoblasts for bone repair.

## Methodology

### Cell culture

This study was approved by the ethics committees of Tokyo Dental College (approval no. 575). Peripheral blood cells were collected from a 32-year-old healthy person who provided informed consent. From these peripheral blood cells, MNCs were selected. Subsequently, T cells were selectively obtained from these MNCs. The T cells were reprogrammed by infection with the Sendai viral vector (containing *OCT3/4*, *SOX2*, *KLF4*, and *c-MYC* genes), as described previously.^19^ After the ES cell-like colonies appeared, the colonies were picked and passaged several times.

Established iPSCs were maintained in human ES medium (Dulbecco’s modified Eagle’s medium, nutrient mixture F-12 [DMEM/F-12, Invitrogen, Carlsbad, CA] with 20% knockout serum replacement [Invitrogen, Carlsbad, CA] supplemented with 1× nonessential amino acid solution [Chemicon, Temecula, CA], 2 mM L-glutamine [Chemicon], 1 mM 2-mercaptoethanol [Wako Pure Chemical Industries Ltd., Osaka, Japan], 1% penicillin/streptomycin [Invitrogen], and 5 ng/mL human FGF-2 [ReproCELL Incorporated, Yokohama, Japan]).

The iPSC colonies were detached using a cell scraper and transferred to low-attachment Petri dishes to generate embryoid bodies (EBs). The EBs were maintained in suspension in human ES medium without FGF-2 for 6 days. On day 6, the EBs were treated with 2 mM thiazovivin (Wako Pure Chemical Industries Ltd.) in human ES medium without FGF-2 for 1 h at 37°C, collected, and dissociated in 0.5 mg/mL collagenase type IV (Wako Pure Chemical Industries Ltd.) for 20 min at 37°C, followed by treatment with 0.05% trypsin–EDTA (Invitrogen) for 5 min at 37°C. The cell suspensions were rinsed with α-MEM (Invitrogen, Carlsbad, CA) with 10% FBS, and the cells were seeded onto culture dishes and cultured in osteoblast medium (OBM; α-MEM with 10% FBS, 50 mg/mL L-ascorbic acid [Wako Pure Chemical Industries Ltd.], 10 mM β-glycerophosphate [Wako Pure Chemical Industries Ltd.]. and 10 nM dexamethasone [Wako Pure Chemical Industries Ltd.]) for 14 days. FGF-2 (25 ng/mL), IGF-1(100 ng/mL), and TGF-β(1 ng/mL) were added on the following day and refreshed every 3 days.^18^

### RNA isolation, reverse transcription (RT) polymerase chain reaction (PCR), and quantitative PCR (qPCR)

**Figure 1:**
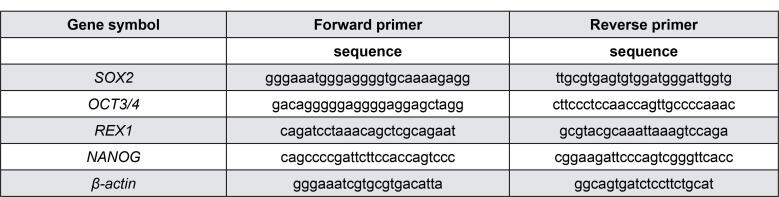
Primers used for PCR

Total RNA was extracted using QIAzol reagent (Qiagen, Hilden, Germany), according to the manufacturer’s instructions. Complementary DNA (cDNA) was synthesized using a high-capacity cDNA RT kit (Thermo Fisher Scientific, city, US state code, country). To confirm the expression of ESC markers, PCR was performed on cDNA with Ex-Taq polymerase (Takara Bio, Inc., Shiga, Japan); *β-actin* was used as an internal control. All primer sets are described in Figure 1.

**Figure 2:**
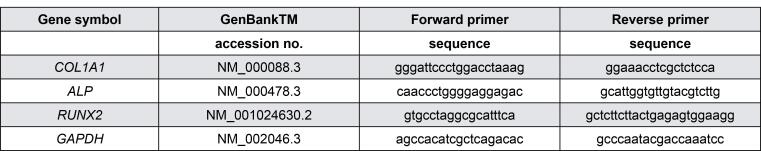
Primers used for RT-qPCR

RT-qPCR was performed using the Premix Ex-Taq reagent (Takara Bio, Inc.) with cDNA, according to the manufacturer’s instructions. Target genes included type 1 collagen (*COL1A1*), tissue nonspecific alkaline phosphatase (*ALP*), and runt-related transcription actor 2 (*RUNX2*); *GAPDH* was used as an internal control. All primers and probes are shown in Figure 2. The relative expression of genes of interest was estimated using the ΔΔCt method.

### Teratoma formation

Human iPSC colonies were removed from the culture plates using a cell scraper. The cells (1 × 10⁶ in 20 μL phosphate-buffered saline) were injected into the testes of 10-week-old male C.B-17 SCID mice (Charles River Laboratories Japan, Inc., Yokohama, Japan). Teratomas were collected 12 weeks after the injections and fixed with 10% neutral buffered formalin for 24 h, paraffin-embedded, and then sectioned for histological assays. All the animal studies were conducted in accordance with protocols approved by the Animal Research Committee of Tokyo Dental College (approval no. 290401).

### Transplantation of iPSCs-OBs

After 4 weeks of osteoblast differentiation, iPSCs-osteoblasts were dissociated with 0.5 mg/mL collagenase type IV for 30 min and 0.25% trypsin–EDTA for 5 min at 37^0^C. These cells (2 × 10^5^) were transplanted in atelocollagen (AteloCell^®^, Atelocollagen honeycomb sponge, KOKEN, Tokyo, Japan) as a scaffold for the cells. Ten-week-old male F344/NJclrnu/rnu rats were obtained from Clea Japan, Inc. (Tokyo, Japan). After anesthesia induction with 4% sevoflurane (Maruishi Pharmaceutical Co. Ltd., Osaka, Japan), the rats were further anesthetized by intraperitoneal injection with sodium pentobarbital (30 mg/kg body weight; somnopentyl; Kyoritsu Seiyaku, Tokyo, Japan). After the calvarial bone was exposed, critical-sized bone defects (diameter = 5 mm) were created in the dorsal area. The scaffold/iPSC–osteoblast complex was transplanted into the bone defects. After transplantation, the periosteal flap was closed by suture.^20^ The rats receiving transplantation were euthanized at 4 weeks and assessed using radiographical and histological analyses.

### Radiographical and histological assessments

Micro-computed tomography (CT) parameters were as follows: X-ray source, 90 kV/100 μA; rotation, 360°; exposure time, 17 s; and voxel size, 50 × 50 × 50 μm (R-μCT^®^; Rigaku Corporation, Tokyo, Japan). Three-dimensional images were constructed using the TRI/3D-BON system (Ratoc System Engineering Co. Ltd., Tokyo, Japan). Sagittal sections (thickness = 5 μm) through the center of each circular defect were prepared and stained with hematoxylin and eosin (H–E) and Masson’s trichrome.

### Statistical analyses

All data are expressed as the mean + standard deviation (SD). Comparisons between each experimental group were performed using the Wilcoxon rank sum test. The statistical significance was defined as p<0.05.

## Results

### iPSC generation from peripheral blood T cells

**Figure 3:**
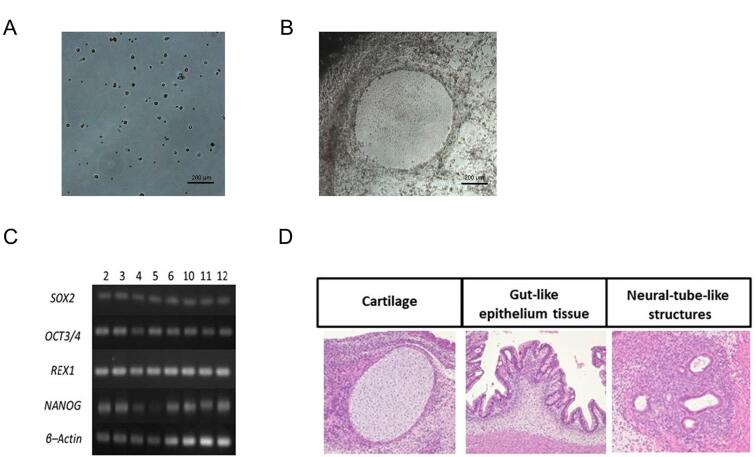
Establishment of iPSCs derived from human peripheral blood T cells

We isolated MNCs from peripheral blood and then obtained T cells selectively from these MNCs (Figure 3A). Embryonic stem-like cells appeared approximately 20 days after the Sendai virus transfection (Figure 3B), which was faster than using human fibroblast cells derived from oral tissues (data not shown). These clones expressed mRNA for undifferentiated markers *SOX2*, *OCT3/4*, *REX1*, and *NANOG in vitro* (Figure 3C). In the teratoma formation assay, tumors were collected approximately 12 weeks after the injections. Histological examinations in the teratoma assay demonstrated that tissues originating from all three embryonic germ layers were present, including cartilage (mesoderm), gut-like epithelium (endoderm), and neural tube–like structures (ectoderm) (Figure 3D).

T cells were obtained selectively from peripheral blood (A). T cells were transfected with the Sendai virus vector SeVdp (KOSM302L). iPSC colonies were selected approximately 20 days after transfection and several clones were subsequently expanded (B). RT-PCR analysis of ESC marker gene expression in several established iPSC clones; β-actin was used as a loading control. The target genes evaluated included SOX2, OCT3/4, REX1, and NANOG (C). Histological examinations in teratoma assays demonstrated that tissues originating from all three embryonic germ layers were present, including cartilage (mesoderm), gut-like epithelium (endoderm), and neural tube–like structures (ectoderm) (D).

### *In vitro* osteoblast differentiation of iPSCs

**Figure 4:**
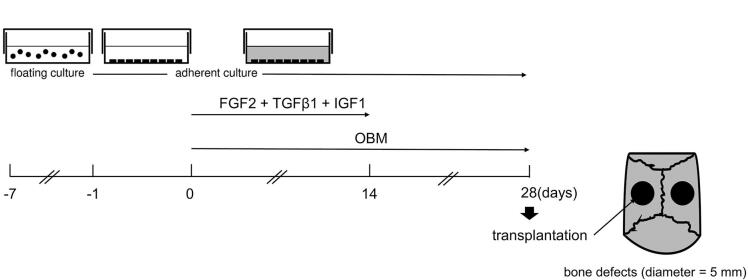
Outline of the protocol for the differentiation of iPSCs into osteoblasts

Osteoblast differentiation from iPSCs was performed as described previously^18^. After EBs were separated into single cells and cultured for adherence, MSC-like cells were found on dishes (Figure 4).

The iPSC colonies were dissociated with a cell scraper and transferred to low-attachment Petri dishes to generate embryoid bodies (EBs). EBs were maintained in suspension in human ES medium without FGF-2 for 6 days. EBs were then separated and seeded into new dishes. Single cells were cultured in OBM using FGF-2, IGF-1, and TGF-β1 for the first half period. After 28 days of osteoblast culture, induced cells were transplanted into rat calvaria bone defects.

**Figure 5:**
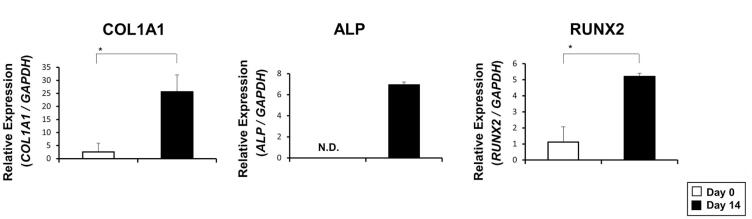
Expression of osteoblast markers after OBM culture

Additionally, osteoblast-like cell migrations could be observed after 14 days of culture. The expression of osteoblast-specific markers was then estimated to confirm the osteoblast differentiation. As expected, osteoblast-specific markers *COL1A1*, *ALP*, and *RUNX2* were upregulated markedly in 14 days in the OBM culture group (Figure 5).

Expression of COL1A1, TNAP, and RUNX2 analyzed using RT-qPCR. mRNA levels were normalized to those of 18S rRNA. Experiments were performed in triplicate. Values represent the mean ± SD, n = 3. Wilcoxon’s rank sum test was applied for comparisons; *p<0.05.

### *In vivo* bone formation

**Figure 6:**
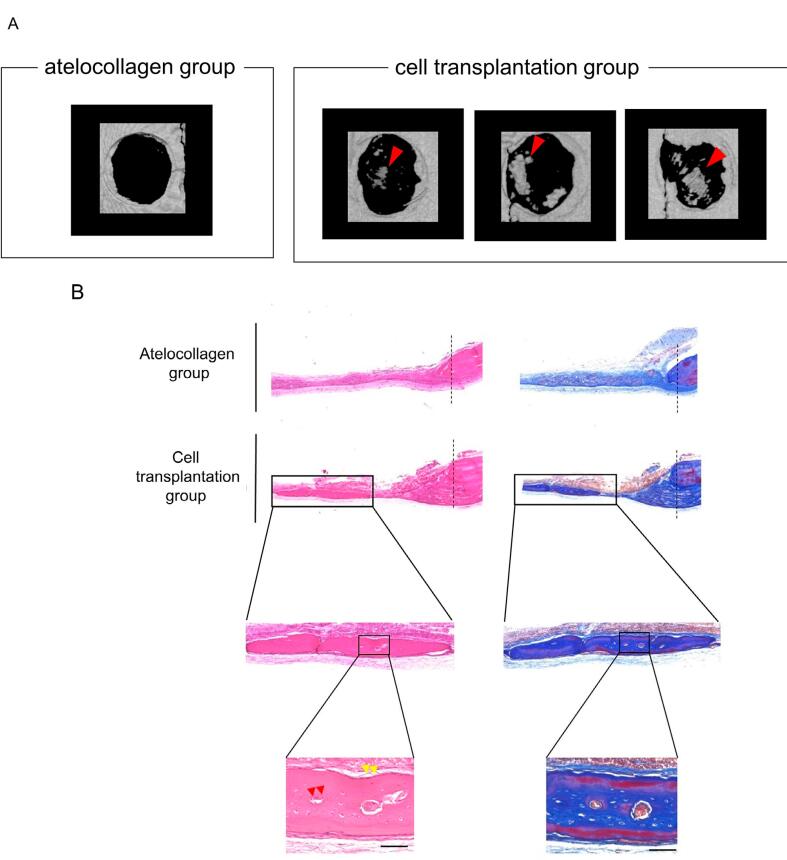
Transplantation of induced osteoblasts and new bone formation

Micro-CT images of the rat calvaria 4 weeks after transplantation are shown in Figure 6A. Newly formed bones were observed in the transplantation group compared with those in the control group 4 weeks after surgery. In addition, we confirmed the bone formation not only from the margin but also scattered in the whole bone defects.

(A) Micro-CT images of the calvaria 4 weeks after transplantation in the cell transplantation and control groups (n = 3). Red arrowheads show new bone formations.

(B) Staining images (left: H–E and right: Masson’s trichrome stain) 4 weeks after cell transplantation or that with atelocollagen scaffold only. The dotted line indicates the cut aspect. Black squares indicate representative parts of newly formed bones. Red arrowheads show osteocytes and yellow arrowheads show the cell lining. Scale bar: 100 µm.

Images of the calvarial histology in coronal H–E and Masson’s trichrome stained sections are shown in Figure 6B. In the control group, osteocytes were lost from the cut area and partially new bone formation was observed only from the margin. However, in the cell transplantation group, new bone formation was observed not only from the margin but also from the central part, and there were cells lining the surface of the new bones and osteocytes inside these; a lamellar structure was also present within these bones. Therefore, these bones are mature and living bones. In addition, a histological analysis revealed that no tumor formation had occurred.

## Discussion

iPSCs are expected to provide both new opportunities for the elucidation of disease mechanisms and personalized regenerative cell therapies, owing to their excellent proliferative ability and pluripotency.^14,21,22^ Recent studies have demonstrated that iPSCs can be generated from different somatic cells and induced to form various types of cells.^23^ Therefore, iPSCs are expected to lead to the advancement of regenerative therapies. Naturally, bone marrow MSCs are the best cells for bone regeneration therapy, but their collection is highly invasive and their performance deteriorates with age. Therefore, the use of iPS cells for bone regenerative therapy is of value.

In this study, we established iPSCs from peripheral blood T cells using the Sendai virus vector and induced the formation of osteoblasts. Although iPSCs are often established from skin fibroblasts, harvesting peripheral blood cells is more convenient and less invasive than harvesting dermal fibroblasts. In addition, T cells are most abundant in peripheral blood cells. If osteoblasts can be induced from peripheral blood cells, this would allow the application of regenerative treatment to adults and children. Furthermore, the use of peripheral blood T cells shortens the cell culture time required to prepare and prime the target cells for reprogramming as compared with fibroblasts.^11^

We used the Sendai virus vector to reprogram MNCs into iPSCs. Unlike other viral vectors, this vector is characterized by its ability to remain in the cytoplasm as RNA and to be replicated, transcribed, and translated there. This avoids the possible integration of vectors into the DNA sequence of the host, preventing damage to chromosomes or the induction of carcinogenesis. In addition, it has been found that the use of the Sendai virus vector can establish iPSCs more efficiently than electroporation or the use of other virus vectors. Thus, the Sendai virus vector is a potential clinical tool for iPSC-based medicine.^19^

We have previously reported on osteoblast induction from human iPSCs.^18^ In a previous study, we reported efficient generation of osteoblastic cells from a human iPSC cell line and subsequent osteogenic induction using vitamin D. However, it was not known whether osteoblasts could be induced from blood cell-derived iPSCs. In this study, iPSCs generated from peripheral blood cells exhibited osteogenic potential after 14 days of induction. The results showed increased osteoblast marker expression, including *COL1A*, *ALP*, and *RUNX2*, which are essential factors for mineralization. Moreover, the transplantation of induced osteoblasts in the collagen sponge accelerated new bone formation in rat critical sized calvaria bone defects. In this study, we confirmed bone formation not only at the margin but also scattered throughout the whole bone defects. This indicated that not only did the transplanted cells induce bone formation at the margin but also formed new bones. In addition, the newly formed bones had a layered structure, and osteocytes were observed inside them, indicating that the bones were functional. However, compared with previous reports, less bone formation was observed in this study. This may be due to the scaffold; therefore, it is necessary to determine the ideal scaffold for osteoblasts derived from iPSCs in the future. Although no tumorigenic lesions were formed in this study, it is necessary to investigate whether tumorigenesis may occur in the longer term.

Here we established iPSCs and induced osteoblasts using human peripheral blood cells, suggesting their usefulness as a cell source for new minimally invasive bone regeneration therapy. However, iPSCs are difficult to handle and induction procedures may not be successful even with the same cells; therefore, it is necessary to increase the number of samples and conduct further studies in advance for clinical applications. This animal experimental model used the relatively easy-to-handle rat skull; however, it will also be necessary to examine a jawbone defect animal model.

Current bone tissue engineering strategies employ combinations of osteoinductive biomaterials, growth factors, and stem cells.^24-26^ As the bone tissue engineering field grows, improved combinations of scaffolding biomaterials and bioreactors will be found, creating a more suitable stem cell microenvironment for new tissue formation. It is said that a three-dimensional porous scaffold having an average pore size ranging between 5 and 20 nm and which is able to support a good cell adhesion is necessary for new tissue formation. In addition, recent studies have reported that new porous scaffolding materials developed using CAD technology and surface treatment of biomaterials were effective for stem cell adhesion and proliferation.^27,28^ In this study, we used collagen sponge as a scaffold to evaluate the ability of MNC-iPSCs to form bone. Therefore, we still need to investigate the ideal osteogenic biomaterials for the induced osteoblasts to apply in a three-dimensional microenvironment that is much larger than the size of cells.

To our knowledge, this is the first study to report that the transplantation of iPSC-derived peripheral blood cells can contribute to new bone formation. This indicates that iPSCs derived from peripheral blood MNCs could be new tools for personalized bone regeneration therapy.

## Conclusion

In this study, we established iPSCs from peripheral blood T cells using the Sendai virus vector and induced the formation of osteoblasts. Although there are some hurdles in iPSC transplantation, our findings may contribute to personalized bone regeneration therapy in the future.
